# Evaluating the Impact of Prevention of Mother-to-Child Transmission of HIV in Malawi through Immunization Clinic-Based Surveillance

**DOI:** 10.1371/journal.pone.0100741

**Published:** 2014-06-26

**Authors:** Michele A. Sinunu, Erik J. Schouten, Nellie Wadonda-Kabondo, Enock Kajawo, Michael Eliya, Kundai Moyo, Frank Chimbwandira, Lee Strunin, Scott E. Kellerman

**Affiliations:** 1 Boston University School of Public Health, Boston, Massachusetts, United States of America; 2 Management Sciences for Health, Lilongwe, Malawi; 3 Ministry of Health, Lilongwe, Malawi; Indiana University and Moi University, United States of America

## Abstract

**Background:**

Prevention of mother-to-child transmission of HIV (PMTCT) programs can greatly reduce the vertical transmission rate (VTR) of HIV, and Malawi is expanding PMTCT access by offering HIV-infected pregnant women life-long antiretroviral therapy (Option B+). There is currently no empirical data on the effectiveness of Malawian PMTCT programs. This study describes a surveillance approach to obtain population-based estimates of the VTR of infants <3 months of age in Malawi immediately after the adoption of Option B+.

**Methods and Findings:**

A sample of caregivers and infants <3 months from 53 randomly chosen immunization clinics in 4 districts were enrolled. Infant dried blood spot (DBS) samples were tested for HIV exposure with an antibody test to determine maternal seropositivity. Positive samples were further tested using DNA PCR to determine infant infection status and VTR. Caregivers were surveyed about maternal receipt of PMTCT services. Of the 5,068 DBS samples, 764 were ELISA positive indicating 15.1% (14.1–16.1%) of mothers were HIV-infected and passed antibodies to their infant. Sixty-five of the ELISA-positive samples tested positive by DNA PCR, indicating a vertical transmission rate of 8.5% (6.6–10.7%). Survey data indicates 64.8% of HIV-infected mothers and 46.9% of HIV-exposed infants received some form of antiretroviral prophylaxis. Results do not include the entire breastfeeding period which extends to almost 2 years in Malawi.

**Conclusions:**

The observed VTR was lower than expected given earlier modeled estimates, suggesting that Malawi’s PMTCT program has been successful at averting perinatal HIV transmission. Challenges to full implementation of PMTCT remain, particularly around low reported antiretroviral prophylaxis. This approach is a useful surveillance tool to assess changes in PMTCT effectiveness as Option B+ is scaled-up, and can be expanded to track programming effectiveness for young infants over time in Malawi and elsewhere.

## Introduction

In 2011, 330,000 children were infected with HIV, with more than 90% of these children living in sub-Saharan Africa [1]. Transmission of HIV from mother to child remains the primary mode of infection in children [2], and prevention of mother-to-child transmission of HIV (PMTCT) programs are the primary strategy to decrease vertical transmission of HIV. Approaches to evaluating the success of PMTCT programs have relied on mathematical models rather than empirical data collection, or measured the implementation of individual PMTCT components for an understanding of program effectiveness (e.g., attendance at antenatal care clinics, HIV-testing for pregnant women, provision of prophylaxis for mother and infant, and safer feeding practices) with little information available on the impact on HIV transmission.

Coverage evaluations have likely produced overly optimistic assessments of PMTCT effectiveness because studies have found coverage measures overestimate PMTCT participation rates [3], [4]; HIV-infected pregnant women dispensed prophylactic antiretrovirals during pregnancy and labor may not actually ingest them; clinic record keeping may be poor; or loss-to-follow-up of mothers and infants may bias results [3], [5], [6]. Alternatively, examination of routine surveillance data by definition excludes women who never accessed antenatal care (ANC) and never realize the benefits of PMTCT. Consequently, the actual vertical transmission rate (VTR) remains unknown in most countries. To understand the true reach and impact of current PMTCT programming, it is critical to utilize methods that capture standardized outcome data on all children prenatally exposed to HIV, not just those who access PMTCT programs.

Malawi has made significant progress in implementing PMTCT since programming was launched in 2003, with PMTCT service sites increasing from 357 in 2007 to 585 in 2012 [7]. PMTCT is fully integrated into maternal and child care, although there has been limited evaluation of the program’s effectiveness. In 2011 the Malawian government was the first to adopt the ‘Option B+’ strategy, offering all HIV-infected pregnant and breastfeeding women antiretroviral therapy (ART) for life, regardless of clinical stage or CD4 count [8]. Two months after the adoption of Option B+ but prior to wide-spread scale-up, we undertook an evaluation of Malawi’s PMTCT program to derive population-based estimates of the VTR by testing a sample of infants presenting for their first immunization clinic visit in four districts. By comparing this estimate of VTR to the results of subsequent evaluations of the national program now that Option B+ is the established PMTCT strategy in Malawi, we will be able to assess trends in the effectiveness of the national PMTCT program for young infants (<3 months), and by extension, the effectiveness of Option B+ as implemented in this setting. In addition this study demonstrates the potential for routine testing at immunization clinics to monitor mother-to-child transmission (MTCT) rates in Malawi, and illustrates the feasibility of this approach for regular data collection for surveillance purposes.

## Methods

The study was reviewed and approved by the Malawi National Health Sciences Research Committee and the Boston University Medical Center Institutional Review Board (IRB). Written informed consent was obtained from all participants; caregivers provided written consent for both their and the infant’s participation. The informed consent form was approved by both reviewing IRBs.

**Figure 1:**
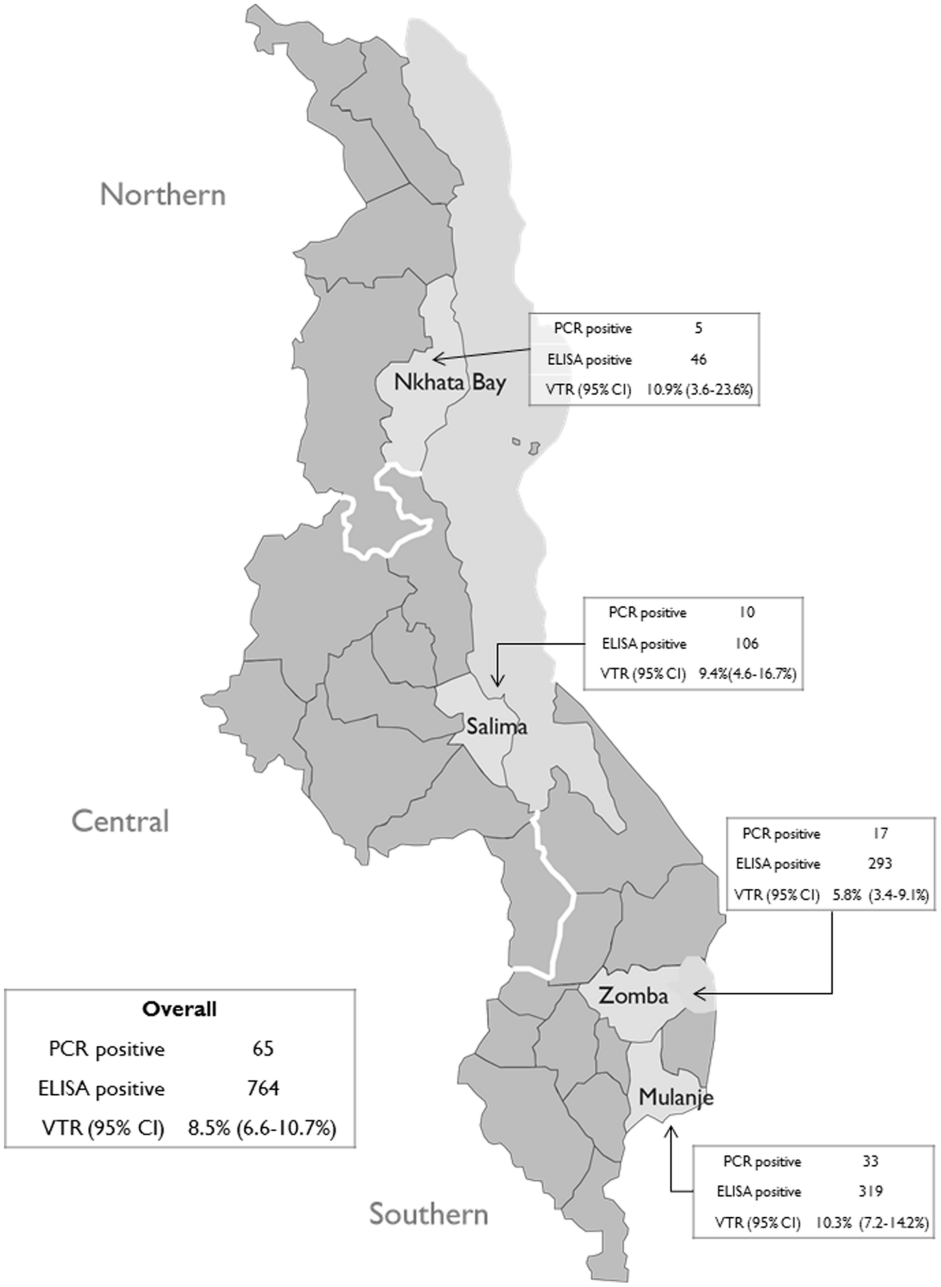
Transmission Rates Overall and by District.

Between September and November 2011 we evaluated the national PMTCT program in four Malawi districts, adapting a surveillance approach based in under-5 clinics developed in South Africa [9], [10]. We tested dried blood spot (DBS) samples from infants <3 months of age presenting for their first immunization visit for maternal HIV antibodies and subsequently for HIV with DNA polymerase chain reaction (PCR) to calculate a population-based HIV VTR. As almost all HIV exposed infants will test positive for maternal HIV antibodies below 3 months of age even if uninfected, the VTR can be estimated as the fraction of antibody positive infants with a reactive DNA PCR (indicating HIV infection). Caregiver-infant pairs were sampled through a three-stage cluster design. The first stage purposively sampled four of the 28 Malawi districts to reflect regional diversity. Districts were sampled proportionate to HIV prevalence, and as HIV prevalence is twice as high in the Southern region (17.6% compared to 8.2% and 9.0% in the North and Central regions respectively [11]), twice as many districts were chosen in that region. Sampled districts were Nkhata Bay in the Northern region, Salima in the Central region, and Mulanje and Zomba in the Southern region (Figure 1). The second stage randomly sampled 53 health facilities within the four districts. The health facilities were sampled proportionate to district population size and stratified by urban and rural location. In the third stage, all infant-caregiver pairs meeting the inclusion criteria at the selected facilities between September and November 2011 were invited to participate.

Infants were required to be less than 3 months of age to ensure detection of maternal antibodies, if present, as a marker of maternal HIV-infected status and neonatal HIV-exposure. In addition, the infant must have presented to the clinic for her or his first immunization (a pentavalent diphtheria, tetanus, pertussis, Haemophilus influenza b, and hepatitis B vaccine) scheduled for 6 weeks of age. The study approach depends on high-levels of participation in the immunization program to ensure a reasonable approximation of the total population of mother-infant pairs in Malawi. The Malawi infant immunization program reports first immunization rates surpassing 97% [11]. In addition, we required the caregiver be in a position to provide consent for biological data to be collected from the infant. Appropriate caregiver roles include parent or legal guardian. Infants were excluded if the caregiver was younger than 18 years of age. Based on an estimated 2010 HIV prevalence rate of 11% for women aged 15–49 [12], an estimated transmission rate of 13.8% [13] and an alpha-level of 5%, our sample size target was approximately 5,500 caregiver-infant pairs to obtain a sample of 600 HIV-exposed infants.

Existing clinic staff were trained to collect study data. After obtaining informed consent, DBS samples were collected via heel stick from infants, and caregivers were surveyed about receipt of maternal HIV testing and PMTCT services. Additional data were collected to determine the feasibility of this surveillance approach, including detailed implementation information from data collectors during supervision visits. Study staff conducted supervision visits to all participating health facilities every two weeks to pick up collected data, conduct quality control activities, and provide additional training as needed.

DBS samples were sent to the Ministry of Health National HIV Reference Laboratory in Lilongwe and tested for maternal HIV antibodies using an enzyme-linked immunosorbent assay (ELISA) test (Vironostika HIV Uni-Form II Ag/Ab BioMerieux, The Netherlands). If positive (indicating maternal HIV infection), the sample was transferred to the University of North Carolina (UNC) laboratory in Lilongwe to be tested for HIV-1 DNA using a PCR test (Amplicor HIV-1 DNA PCR V1.5 Roche) to determine the infant’s HIV status. Individual test results were returned to clinics to be provided to infant caregivers at the subsequent infant immunization visit.

We calculated population and district level VTR estimates and conducted analyses describing characteristics of PMTCT participation for mothers with ELISA positive infants. Estimates were calculated using Generalized Estimating Equations to account for the clustered nature of the data. All analyses were undertaken using SAS software version 9.3 [14].

## Results

Data were collected on 5,634 caregiver-infant pairs. Participants were excluded (not mutually exclusive) because they did not fit the inclusion criteria (age of infant (6 excluded), age of caregiver (325 excluded), not presenting for first vaccination (113 excluded), and relationship to the infant (4 excluded)). An additional 88 caregiver-infant pairs were excluded due to invalid DBS sample (e.g. insufficient amount of blood provided), 80 were excluded due to missing DBS sample, and 10 were excluded due to missing survey data. Forty-four mothers self-reported positive HIV status on the survey, but their infant’s DBS sample tested ELISA negative. We did not perform DNA PCR testing on these samples and, as we could not ascertain the reason for the discrepancy, these 44 caregiver-infant pairs were not included in the analysis as HIV-infected or HIV exposed. The linked survey and sample data for 5,068 caregiver-infant pairs were used in the analysis. We enrolled fewer eligible caregiver-infant pairs than targeted, but our sample included a larger number of exposed infants than required by our sample size calculation.

Eligible caregivers included parents and legal guardians 18 years and older, however the majority of caregivers (98.2%) were the infant’s mother. The average caregiver age was 25 years old, and the average infant age was 8 weeks. The majority of participating caregivers reported maternal ANC attendance (98.4%) and previous maternal HIV testing (95.3%).

Of the 5,068 DBS samples included in the analysis 764 were ELISA positive, indicating 15.1% (14.1–16.1%) of mothers were HIV-infected and had passed HIV antibodies to their infant. Sixty-five of the 764 ELISA-positive samples tested positive with DNA PCR, indicating a vertical transmission rate of 8.5% (6.6–10.7%) across the four districts. There was some variation between districts, with Zomba recording the lowest transmission rate at 5.8% and Nkhata Bay recording the highest at 10.9%; rates in Salima and Mulanje were similar to the rate in Nkhata Bay (9.4% and 10.3% respectively) (Figure 1).

The majority of HIV-infected mothers (as defined by their infant’s positive ELISA result) self-reported receiving some PMTCT services (Table 1). Most HIV-infected mothers (98.2%) reported attending ANC at least once during their pregnancy, with similarly high proportions in all four districts. Most HIV-infected mothers also reported testing for HIV (94.9%) and over 70% reported previously testing positive, indicating most, but not all HIV-infected women knew, or were willing to share their serostatus. One hundred seventy nine (23.4%) infected mothers reported their most recent HIV test was negative, suggesting some women recently seroconverted, received a previous false negative result or a false positive ELISA result, or chose not to share their seropositive status with the data collectors. In Nkhata Bay a larger proportion of ELISA positive mothers reported never testing (6.5%) compared to other districts (0.0–3.5%), and a smaller proportion knew their HIV status (63.0%) compared to other districts (over 70% in each of the other three districts). At the time of data collection, 92.5% of exposed infants (all younger than 3 months) were exclusively breastfed.

**Table 1:** Self-Reported Participation in PMTCT Activities among HIV-infected Mothers.

	% Total (#)	% Nkhata Bay	% Salima	% Zomba	% Mulanje
	N = 764	N = 46	N = 106	N = 293	N = 319
Mother attended ANC at least once					
Yes	98.2 (750)	95.7 (44)	100.0 (106)	98.0 (287)	98.1 (313)
No	0.7 (5)	2.2 (1)	0.0 (0)	0.7 (2)	0.6 (2)
Missing	1.2 (9)	2.2 (1)	0.0 (0)	1.4 (2)	1.3 (4)
Mother’s Most Recent HIV Test Result^1^					
Never tested	2.1 (16)	6.5 (3)	0.0 (0)	0.7 (2)	3.5 (11)
Negative	23.4 (179)	26.1 (12)	26.4 (28)	22.2 (65)	23.2 (74)
Positive	71.5 (546)	63.0 (29)	70.8 (75)	73.4 (215)	71.2 (227)
Don’t know/Missing	3.0 (23)	2.3 (2)	2.8 (3)	3.8 (11)	2.2 (7)
Mother Received ART During Pregnancy and/or Labor					
Yes	64.8 (495)	58.7 (27)	62.3 (66)	65.9 (193)	65.5 (209)
No	30.1 (230)	39.1 (18)	35.9 (38)	27.3 (80)	29.5 (94)
Missing	5.1 (39)	2.2 (1)	1.9 (2)	6.8 (20)	5.0 (16)
Infant Received ART Prophylaxis					
Yes	46.9 (358)	45.7 (21)	52.8 (56)	52.2 (153)	40.1 (128)
No	46.5 (355)	45.7 (21)	45.3 (48)	39.3 (115)	53.6 (171)
Missing	6.7 (51)	8.7 (4)	1.9 (2)	8.5 (25)	6.3 (20)
Exclusive Breastfeeding					
Yes	92.5 (707)	87.0 (40)	93.4 (99)	91.1 (267)	94.4 (301)
No	6.2 (47)	10.9 (5)	6.6 (7)	7.9 (23)	3.8 (12)
Missing	1.3 (10)	2.2 (1)	0.0 (0)	1.0 (3)	1.9 (6)

1 Caregiver report prior to ELISA testing.

The provision of antiretroviral (ARV) drugs to HIV-infected pregnant women and exposed infants is critical for the success of PMTCT. In Malawi, the current treatment regimen for pregnant women under Option B+ is Tenofovir, Lamivudine, and Efavirenz in a daily fixed-dose single tablet [8]. The HIV-infected mothers in our study reported receiving several different ARV regimens with additional variation across districts. During pregnancy 38.6% reported not receiving any ARV prophylaxis (Table 2). By district this finding ranged from 35.4% in Mulanje to 44.3% in Salima.

**Table 2:** HIV Treatment Regimen during Pregnancy.

	% Total (#)	% Nkhata Bay	% Salima	% Zomba	% Mulanje
Treatment Regimen	N = 764	N = 46	N = 106	N = 293	N = 319
None	38.6 (295)	43.5 (20)	44.3 (47)	39.3 (115)	35.4 (113)
AZT	29.3 (224)	21.7 (10)	17.0 (18)	20.1 (59)	42.9 (137)
ART^2^	26.1 (199)	30.4 (14)	34.9 (37)	33.8 (99)	15.4 (49)
Don’t know/Missing	6.1 (46)	4.4 (2)	3.8 (4)	6.8 (20)	6.3 (20)

2 Any of the first or second line regimens used in Malawi at the time, including: D4T+3TC+NVP, d4T+3TC/EFV, AZT+3TC/EFV, AZT+3TC/TDF/LPV/r.

When asked whether they received ARVs during labor, one-third reported receiving none (Table 3). By district, the percent of mothers who reported not receiving ARVs ranged from 29.4% in Mulanje to 41.3% in Nkhata Bay. Among women receiving ARVs, similar proportions took single dose Nevirapine (18.2%), Nevirapine/AZT/3TC (22.3%), and ART, defined as any of the first or second line regimens used in Malawi at the time (20.4%), with some variations across districts.

**Table 3:** HIV Treatment Regimen During Labor.

	% Total (#)	% Nkhata Bay	% Salima	% Zomba	% Mulanje
Treatment Regimen	N = 764	N = 46	N = 106	N = 293	N = 319
None	33.5 (256)	41.3 (19)	37.7 (40)	34.8 (111)	29.4 (86)
Single-dose Nevirapine (NVP)	18.2 (139)	6.5 (3)	22.6 (24)	19.8 (63)	16.7 (49)
NVP AZT 3TC	22.3 (170)	21.7 (10)	17.0 (18)	28.2 (90)	17.8 (52)
ART^3^	20.4 (156)	28.3 (13)	21.7 (23)	11.9 (38)	28.0 (82)
Don’t Know/Missing	5.6 (43)	2.2 (1)	0.9 (1)	5.3 (17)	8.2 (24)

3 Any of the first or second line regimens used in Malawi at the time, including: D4T+3TC+NVP, d4T+3TC/EFV, AZT+3TC/EFV, AZT+3TC/TDF/LPV/r.

In total, 64.8% of HIV-infected mothers reported receiving some form of ARV prophylaxis during pregnancy and/or labor. A smaller proportion of exposed infants received treatment with caregivers reporting less than half of exposed infants received ARV prophylaxis (46.9%) (Table 1). Recommended infant ARV regimens in Malawi at the time of data collection were single dose Nevirapine, single dose Nevirapine followed by 1–4 weeks of AZT syrup, or 1–4 weeks of AZT syrup only.

Table 4 presents the number and proportion of PCR positive infants over ELISA positive infants, and the relative risk (RR) of transmission for factors associated with vertical transmission. Transmission rates were proportionally higher among young mothers (aged 18–20), mothers who reported not attending ANC, delivered at home and/or with a Traditional Birth Attendant (TBA), never tested for HIV, did not receive HIV treatment during pregnancy or labor, and whose children did not receive prophylactic ARVs; however these results were not statistically significant. Infants who did not receive prophylactic ARVs after birth had a marginally significant increased risk of transmission.

**Table 4:** Relative Risk of HIV Transmission by Risk Factor Among ELISA Positive Infants.

Variables	# of PCR-pos^4^(% of ELISA-pos)	Relative Risk(95% CI)	P-value
Age of Mother			
18–20 years	9 (12.5)	1.5 (0.8–2.9)	0.19
21–30 years (reference)	36 (8.1)		
31–40 years	20 (8.6)	1.0 (0.7–1.7)	0.84
Mother Attended ANC			
Yes (reference)	63 (8.4)		
No	1 (20.0)	2.4 (0.4–15.4)	0.36
Place of Delivery			
Hospital or Clinic (reference)	53 (8.2)		
Home and/or with Traditional Birth Attendant	12 (11.8)	1.4 (0.8–2.4)	0.18
Mother’s Most Recent HIV Test Result^5^			
Positive (reference)	41 (7.5)		
Never tested	3 (18.8)	2.5 (0.8–7.7)	0.11
Negative	20 (11.2)	1.5 (0.9–2.6)	0.15
Mother Received HIV Treatment During Pregnancy and/or Labor			
Yes (reference)	37 (7.5)		
No	24 (10.4)	1.4 (0.9–2.2)	0.15
Infant Received Prophylactic ARVs			
Yes (reference)	24 (6.7)		
No	37 (10.4)	1.6 (1.0–2.5)	0.07

4 N = 65– “don’t know” and “missing” responses are not presented in the table.

5 Caregiver report prior to ELISA testing.

Data were collected formally and informally throughout implementation to understand the success of the approach, including gathering satisfaction information during data collector training, documenting sample collection and site issues through an evaluation-specific supervision form, and examining the survey and DBS data for quality. There were no specific problems noted by the data collectors, and data were gathered without significant disruption to their clinical duties. Supervision visits ensured data collection was on track and improved the quality of the data collected. Sufficient numbers of eligible caregiver-infant pairs were enrolled in the study within the expected timeframe.

## Discussion

This study found an overall population-based VTR of 8.5% for Malawian infants born between July and October 2011. While lower than previously modeled rates (i.e., 24.7% [15]), our estimate was for transmission up to 3 months of age only (infant average age was 8 weeks) and did not include the majority of the breastfeeding period. If our results are compared to a 20% estimate for perinatal transmission in the absence of intervention [16], it appears that the Malawian PMTCT program is preventing the majority of vertical transmission up to 2–3 months post-partum, despite the fact that Option B+ (launched in July 2011), was not fully rolled out at the time of data collection. However, low reported coverage of ARVs (64.8% for pregnant women and 46.9% for exposed infants) indicates that further improvements could be made through better implementation of PMTCT. Issues related to accessing ARVs in Malawi and similar settings are persistent and well documented, and include limited CD4 cell count measurement infrastructure, poor linkages between HIV testing and treatment, difficulty accessing health facilities, and stigma [17]–[24]. An important effort to reduce barriers to maternal treatment and decrease the complexity of ARV provision is Malawi’s adoption of Option B+. By putting all HIV-infected pregnant and breastfeeding women on ARVs for life, regardless of clinical stage or CD4 count, Option B+ has the potential to simplify access to treatment among HIV-infected pregnant women and greatly reduce transmission of HIV to infants [8], [24]. Approximately one year into implementation, the Ministry of Health in Malawi reported 58% coverage of HIV-infected pregnant women by Option B+ [25]. The data produced by this study provides a baseline to measure the effectiveness of Option B+, and to ensure delivery occurs as anticipated as it continues to be scaled up across Malawi.

Transmission rates varied by district, with similar rates found in Nkhata Bay, Salima, and Mulanje (10.9%, 9.4%, and 10.3% respectively), and a significantly lower rate found in Zomba (5.8%). Nkhata Bay’s marginally higher VTR can be accounted for by lower rates of participation in PMTCT activities compared to the other districts. Although the differences were small, mothers in the more rural Nkhata Bay were more likely to report never testing for HIV and were less likely to report receiving ARVs.

It is not clear why the transmission rate in Zomba was much lower than in the other districts. Mothers in Zomba reported participating in PMTCT activities at a similar rate as mothers in Salima and Mulanje and did not receive a higher proportion of more efficacious ARV regimens. Public and private systems for HIV prevention and treatment are more developed in the South Eastern region (containing Zomba and Mulanje) as evidenced by the proportion of health facilities providing optimal PMTCT services. Immediately prior to the time of data collection 70% of health facilities in the South Eastern region were providing Option B+ compared to 48% of health facilities in the Northern region (containing Nkhata Bay) and 46% of the Central East region (containing Salima) [26]. Other differences in PMTCT, ART, and maternal and child health services by region near the time of data collection were not identified [11]. The lower transmission rate in Zomba may alternatively be the result of the overall low number of transmissions in the evaluation. It may be valuable for future research to investigate differences in transmission rates by district.

The proportion of infants exposed to HIV in our sample (15.1%) is higher than the HIV prevalence rate for women aged 15–49 (12.9%) identified by the 2010 MDHS [11]. Despite this difference, regional variation in MDHS prevalence rates (Northern region: 8.2%, Central region: 9.0%, and Southern region: 17.6%) [11] were similar to regional differences found in our results of maternal prevalence (Nkhata Bay in the Northern region: 9.1%, Salima in the Central region: 10.4%, and Zomba and Mulanje in the Southern region: 17.9% and 16.6% respectively).

High reported rates of ANC attendance at least once during pregnancy among our study population (98.2%) indicate that ANC remains an important venue for HIV-infected women to be identified and linked to care. Although many HIV-infected women already knew their status due to previous testing, we found infant HIV testing at immunization clinics was another critical opportunity for mothers to learn their HIV status as well as that of their children. Of the 5,068 women enrolled in the study 163 (3.2%) reported never testing, of which 16 women were ELISA positive (representing 2% of all HIV-infected women in the study). In addition, 179 mothers identified as HIV-infected reported their HIV status to be negative on the study survey, further suggesting that provision of testing at immunization clinics has the benefit of identifying previously unknown HIV-infected women.

In countries with generalized epidemics like Malawi, sero-surveillance efforts have long included anonymous, unlinked sero-surveys of pregnant women attending ANC clinics to determine prevalence rates among women of childbearing age. However, existing methods cannot gauge the impact of PMTCT programming on vertical transmission, identify prevalence rates among infants, or provide information on those who do not attend ANC. This study successfully demonstrated the feasibility of an immunization clinic approach for surveillance data collection; enabling the use of existing health system structures to improve the approach’s sustainability and scalability. Current health facility staff were employed as data collectors, all laboratory testing was conducted in-country, and the District Health Officers were engaged as partners for implementation. Furthermore, using DBS for sample collection added to implementation feasibility. DBS collection requires few supplies and samples are easily transported for testing as they are light weight and stable. All these elements were identified as important contributors to successful implementation, and immunization-based surveillance may offer a viable alternative to existing ANC anonymous sero-surveillance activities in Malawi and similar settings.

Several important limitations exist in this study. First, despite planning to collect information on refusal rates, these data were not collected. Without this information we do not know how well our study results reflect the population of caregiver-infant pairs who attend immunization clinics. In addition, the lack of refusal rates makes it difficult to draw definitive conclusions about the acceptability of this methodology with our target population. However, regular supervision visits by study staff to all clinic sites did not identify high refusal rates as an issue. Our calculation of relative risk (RR) of factors associated with transmission resulted in effects in the expected direction; however given the small number of HIV-infected infants, p-values were marginal or not statistically significant. The study was not powered to investigate additional differences between subgroups. The self-reported survey data should be interpreted with caution. Finally, this study examines mother-to-child transmission for infants <3 months of age only as the methodology relies on identifying exposed infants through the presence of maternal antibodies at a site in which the majority of children could be accessed. Although maternal antibodies decrease with time [27], [28], infant immunization clinics provide a unique opportunity for evaluating PMTCT outcomes given high immunization rates and the early persistence of maternal antibodies. Repeating this surveillance approach during subsequent immunization visits will allow a better understanding of HIV transmission throughout the breastfeeding period, however the accuracy of VTR estimates will be affected by the fading of maternal antibodies compounded by decreases in immunization clinic attendance as children age [29]. Additional methods are needed to assess transmission beyond the early infant period, such as provider initiated testing in nutrition clinics, sick child visits, and hospital admissions. Such efforts alone will underestimate the VTR as they likely reflect a smaller proportion of the total population, and, without treatment, 50% of HIV-infected infants die before their second birthday [30]. Other approaches have been developed to determine transmission rates through the breastfeeding period and HIV free survival, such as cohort studies, clinic service data, and the PMTCT Effectiveness in Africa: Research and Linkages to Care (PEARL) method which involves a household survey and the collection of infant DBS samples [31], [32]. Despite the measurement of early transmission only, the important benefits of the methodology used in this study are the ease of implementation, the relatively low cost, and the short time frame (approximately 6 months from sampling to conclusion of data analysis), all of which increase the feasibility of repeating this activity to evaluate trends in PMTCT performance over time. Survey and DBS collection require few special supplies and many health facility staff are already familiar with collecting DBS samples for clinical care and Early Infant Diagnosis (EID). We are developing an implementation toolkit, including all training and study materials, to facilitate the replication of the study with fidelity in Malawi and similar settings. Finally, by incorporating sequential yearly or biannual surveys, Ministries of Health, donors and other interested parties can track the success of their PMTCT programming over time, noting the impact of policy and implementation changes, such as the adoption of Option B+.

This novel approach used to evaluate PMTCT and develop a population based VTR was successfully piloted in four Malawi districts. Existing clinic staff accommodated study data collection into their clinical responsibilities with minimal disruption, and study staff supervision visits were important in ensuring high quality data collection. This method can be successfully replicated and scaled up in Malawi to assess the impact of Option B+ and other settings where immunization rates and HIV prevalence are high to assess the impact of PMTCT programming on vertical transmission. This study demonstrates a simple and efficient approach for PMTCT surveillance, and represents a real solution to the problem faced by many countries of assessing the impact of PMTCT programming and investment.
